# The use of solvent-preserved human and bovine cancellous bone blocks for lateral defect augmentation - an experimental controlled study in vivo

**DOI:** 10.1186/s13005-021-00275-1

**Published:** 2021-06-29

**Authors:** Lara Schorn, Tim Fienitz, Kathrin Berndsen, Norbert R. Kübler, Henrik Holtmann, Daniel Rothamel

**Affiliations:** 1 Department of Oral, Maxillofacial and Facial Plastic Surgery, University Hospital Duesseldorf, Moorenstr. 5, 40225 Düsseldorf, Germany; 2 Department of Oral Surgery, University Hospital Duesseldorf, Duesseldorf, Germany; 3 Department of Oral, Maxillofacial and Facial Plastic Surgery, University Hospital Cologne, Cologne, Germany; 4 Department of Oral, Maxillofacial and Facial Plastic Surgery, Evangelisches Krankenhaus Bethesda, Mönchengladbach, Germany

**Keywords:** Lateral bone augmentation, Horizontal defects, Solvent-preserved bone, Xenogeneic bone augmentation, Critical size defects

## Abstract

**Background:**

The aim of this study was to compare new bone formation, resorbed bone matrix, and fibrous enclosed residual bone substitute material in laterally augmented alveolar bone defects using allogeneic, pre-treated and cleaned human bone blocks (tested in dogs, therefore considered to be xenogeneic), and pre-treated and cleaned bovine cancellous bone blocks, both with and without a collagen membrane in order to evaluate their augmentative potential.

**Methods:**

Thirty-two critical size horizontal defects were prepared in the mandible of 4 adult foxhound dogs (8 per dog, 4 on each side). After 3 months of healing, the defects were laterally augmented in a split-mouth-design with either human (HXB) or bovine solvent-preserved bone blocks (BXB). Afterwards, defects were randomly covered with a bovine collagenous membrane (HXB + M, BXB + M). After a healing interval of 6 months, percentages of new bone formation, resorbed bone matrix, and fibrous enclosed residual bone substitute material were compared.

**Results:**

Results showed little new bone formation of up to 3.7 % in human bone blocks (HXB 3.7 % ± 10.2, HXB + M 0.3 %± 0.4, BXB, 0.1 % ± 0.8, BXB + M 2.6 % ± 3.2, *p* = > 0.05). Percentages of fibrous encapsulation were higher in human bone blocks than in bovine bone blocks (HXB 71.2 % ± 8.6, HXB + M 73.71 % ± 10.6, BXB, 60.5 % ± 27.4, BXB + M 52.5 % ± 28.4, *p* = > 0.05). Resorption rates differed from 44.8 % in bovine bone blocks covered with a membrane to 17.4 % in human bone blocks (HXB 17.4 % ± 7.4, HXB + M 25.9 % ± 10.7, BXB, 38.4 % ± 27.2, BXB + M 44.8 % ± 29.6, *p* = > 0.05). The use of additional membranes did not significantly affect results.

**Conclusions:**

Within its limitations, results of this study suggest that solvent-preserved xenogenic human and bovine bone blocks are not suitable for lateral bone augmentation in dogs. Furthermore, defect coverage with a membrane does not positively affect the outcome.

## Background

Tooth loss due to extraction or trauma often results in a vertical and horizontal loss of bone height due to remodeling processes [1]. During this remodeling process, more horizonal bone than vertical bone (29.63 % to 11–22) is lost [2]. One of the reasons might be differences in defect geometry. Three-walled defects present with a better prognosis than single-walled defects. Horizontal single-walled defects are usually particularly difficult for reconstruction and augmentation.

Several augmentative techniques and materials have been described for bone regeneration. Applied methods for reconstruction and augmentation of horizontal bone defects vary according to expertise and preferences of patients and therapists. Options are bone spreading and bone splitting. These, however, require a primary horizontal width of at least 3–4 mm and without any other augmentative treatment underlie similar resorption rates to those after tooth extraction. Disadvantages are long treatment duration, high relapse rates, and possible post-operative complications, such as early, delayed or absent bone consolidation, nerve injury, and infection [3].

Autogenous, allogenic, xenogeneic or alloplastic bone grafts as well as tissue-engineered materials can be used for horizontal bone regeneration. Autogenous bone is the most commonly used and current gold standard [4]. Bone from intraoral donor sites is preferred, autologous bone blocks from extraoral donor sites, such as iliac crest grafts, are used for severely atrophic maxillae [5]. Disadvantages are limited availability and donor site morbidity [6]. In addition, especially for iliac crest grafts, high resorption rates of up to 60 % have been reported [7]. Allogenic bone blocks have proven successful for alveolar ridge augmentation [8]. However, patients and therapists sometimes seem to have reservations when using them because of a formerly described risk of infection [9, 10].

In terms of lateral augmentation, bovine substitutes have successfully been used. They enhance augmentation due to osteoconductive protentional and slow resorption [11]. In dental surgery mostly deproteinized bovine bone (DBB) material is used. Prior to its use, the tissue has to be purified to ensure the removal of immunogenic components and pathogens. One of the many methods of purification is the Tutoplast® process, in which parts of the proteinized matix are preserved by gentle processing. These solvent-preserved (due to solvent preservation, collagen and mineral structures remain intact) cancellous bone blocks promise high osteoconductive properties with great volume stability due to their proteinized matrix (collagen). Human solvent-preserved bone blocks have successfully been used in sinus elevation procedures [12]. Bovine solvent-preserved cancellous bone has so far only been used in orthopaedic surgery [13]. These bone blocks out of rip bones offer a cortical side to protect it from resorption and a spongious side for blood vessel and stem cell ingrowth.

Aim of this study was to compare solvent-preserved human and bovine cancellous bone blocks in their ability to regenerate lateral alveolar bone defects and hypothesized that because of their high proportion in collagen lateral augmentation would be possible. Furthermore, we hypothesized that bovine solvent-preserved bone blocks would be equivalent to human bone solvent-preserved bone blocks in terms of new bone formation. In order to compare both materials under the same conditions dogs were used. Therefore, both had to be considered xenogeneic. We hypothesized that under the same conditions, there would be no difference in the ability to augment bone. Additionally, in order to create unobstructed conditions, the use of a supplementary collagen membrane was tested for its efficiency when augmenting horizonal defects with solvent-preserved bovine and allogeneic bone blocks.

## Methods

Thirty-two critical size lateral three-walled defects (15 × 10 × 3 mm) were prepared at the mandibula of 4 adult foxhound dogs (4 on each side). The sample size was based on a power analysis including an additional drop-out rate of 5 %. (effect size 1.3, G*Power, Heinrich Heine University, Düsseldorf, Germany [14]). The defects were laterally augmented in a split-mouth-design with either human (HXB) or bovine (BXB) solvent-preserved bone blocks (15 × 10 × 6 mm).

Afterwards, half of the defects were covered with a bovine collagenous membrane for GBR (HXB + M, BXB + M). After a healing interval of 6 months the specimens were harvested and prepared for histological and histomorphometric evaluation. Percentages of new bone formation (NBF), resorbed bone matrix (RM), and fibrous enclosed residual bone substitute material (FE) were compared.

Evaluated groups:

1. HXB: solvent-preserved human bone blocks, Tutogen CS-Block® human, Tutogen Medicals, Neukirchen am Brandt, Germany (*n* = 8).
2. BXB: solvent-preserved bovine bone blocks, Tutogen CS-Block® bovin, Tutogen Medicals, Neukirchen am Brandt, Germany (*n* = 8).
3. HXB + M: solvent-preserved human bone blocks covered with a collagenous membrane out of bovine pericardium, Tutogen CS-Block® human, Tutogen Medicals + Tutodent® Membran, Tutogen Medicals, Neukirchen am Brandt, Germany (*n* = 8).
4. BXB + M: solvent-preserved bovine bone blocks covered with a collagenous membrane out of bovine pericardium, Tutogen CS-Block® bovin + Tutodent® Membran, Tutogen Medicals, Neukirchen am Brandt, Germany (*n* = 8).

Inactivating steps of the purification process of the solvent preserved bone blocks were: cleaning of the tissue with saline solutions of various concentrations, treatment with H2O2 and acetone, sonication in acetone, and a final sterilization via γ-radiation with 17.8 kGy. This offers a loss of antigenicity and viral, bacterial, and prional safety [15]. The allocation of membranes and bone blocks was randomized using randomization software (standard randomization protocol, random.org, Randomness and Integrity Services Ltd, Dublin, Ireland).

### Subjects

Adult foxhound dogs of both genders were treated in this in vivo study (age: 12 months, weight 32 kg). They were kept according to official standards. The use of dogs has been approved by the state office for nature, environment, and consumer protection of North-Rhine Westphalia, Germany (Landesamt für Natur, Umwelt und Verbraucherschutz Nordrhein-Westfalen, LANUV NRW AZ: 50.05-230-7/06). Termination criteria were increase of body temperature over 2 °C for more than 3 days, large wound infections at operating site, immobility, weight loss of over 20 % of former bodyweight. This study was conducted in compliance with the ARRIVE guidelines for animal research.

### Surgical protocol

Two surgical interventions were performed. The first was performed to remove teeth and to create lateral defects. After disinfection and sterile dressings, the molar and premolar teeth of both sides were extracted. By incision and dull preparation, the mandibular bone was exposed and the lateral alveolar bone was cut down to an area of 6 × 2 cm by milling. A multi-layered wound closure was performed by interrupted sutures using Vicryl 4.0. (Vicryl® 4/0, Ethicon GmbH, Norderstedt, Germany). The surgery took 20–35 min. Three months were allowed for healing to transfer the acute into chronical defects.

After a healing period of 3 months, a second surgical intervention was performed to augment the bone with the tested materials. After incision and dull preparation, the mandibular bone was exposed again. Defect sites were smoothened by milling and perforated by drills in order to create sufficient blood supply. Afterwards, bovine and human bone blocks were randomly inserted following a split-mouth-design. Bone blocks were fixed by a single osteosynthetic screw (Straumann GmbH, Waldenström, Germany). At least 3 mm were left in-between the bone blocks. Subsequently, half of the bone blocks were covered randomly by collagenous membranes. A multi-layered wound closure was performed by interrupted sutures using Vicryl 4.0. (Vicryl® 4/0, Ethicon GmbH, Norderstedt, Germany). After 6 months the dogs were euthanized by pentobarbital overdosing (Eutha 77® ad us. vet, Essex Pharma, Muenchen, Germany) and the augmented bone blocks were harvested.

For all surgical procedures animals underwent general anaesthesia. General anaesthesia was induced by Acepromazin (Vetranquil® 1 %, Ceva Tiergesundheit, Düsseldorf, Germany) and 21.5 mg/kg Thiopental-Natrium (Trapanal® 2.5 %, Altana GmbH, Konstanz, Germany), followed by endotracheal intubation. Anaesthesia was maintained by Isoflurane. The operation took around 45–60 min. During surgery and for 4 days postoperatively, the dogs received oral antibiotic coverage (11.0 mg/kg, Clerobe®, Pharmacia Tiergesundheit, Erlangen, Germany). Pain management using 4.5 mg/kg Carprofen (Rimadyl®, Pfitzer Pharma GmbH, Karlsruhe, Germany) started 24 h before the procedure and was continued for 4 days. Additionally, for intraoperative pain management, 0.4 mg/kg Piritramid (Dipidolor®, Janssen-Cilag GmbH, Neuss, Germany) was administered.

### Histological and histomorphometric preparation

Specimens were fixed in 4 % formaldehyde for one week, dehydrated, embedded in Technovit® 7200 VLC (Heraeus Kulzer GmbH, Wehrheim, Germany), and polymerized. Afterwards, samples were cut (Exact 300, EXACT-Apparatebau, Norderstedt, Germany) through the centre of the screws in order to offer equal samples. Thin histological cuts were prepared using grinding machines (Exact 400, EXACT-Apparatebau, Norderstedt, Germany). Samples were stained according to manufacturer’s protocols with Toluidine-blue stain (chosen for histomorphometric quantification). To examine, evaluate, and photograph the specimens a light microscope (Leica DM 5000B, Leica Microsystems, Wetzlar, Germany), equipped with a microscopic high-resolution camera (Leica DFC 40,020 C, Leica Wetzlar, Germany), was used. With the help of image measuring software (SIS AnalySIS Auto Software 3.2, Soft Imaging System) three main measurements were performed: percentages of resorbed augmented bone matrix (RM), percentages of regenerated augmented bone matrix / new bone formation (NBF), and percentages of fibrous encapsulation of bone substitute material (FE). For the measurements, the former bone blocks were, based on the length of the screws for osteosynthesis and the defect size, graphically reconstructed. Afterwards, bone matrix, residual bone material, and new bone formation were color-coded and the difference was calculated. Color-coding of the measurements was manually adjusted when necessary. An investigator well-experienced in histomorphometrical and histological evaluation and blinded to the study conditions performed the measurements.

### Statistical analysis

The methodology was reviewed by an independent statistician. Both a Kolmogorov-Smirnov-Test and a Shapiro-Wilk-Test were used to detect normal distribution of values. Since measurements turned out to be non-parametric, a Mann-Whitney-U-Test was performed for evaluation of dependencies. *P* < 0.05 was set for a significant difference. Calculations were performed using SPSS 22 for Windows (SPSS Inc., Chicago, IL, USA).

## Results

Twenty-seven bone blocks were used for evaluation. Out of the initial 32, four samples were lost due to infection (all four on one side of the jaw, 2x HXB, 2x HXB + M). Additionally, one bovine block (BXB) was lost due to a small wound dehiscence in the augmented area. None of the specimens were rejected.

### Histology

**Fig. 1:**
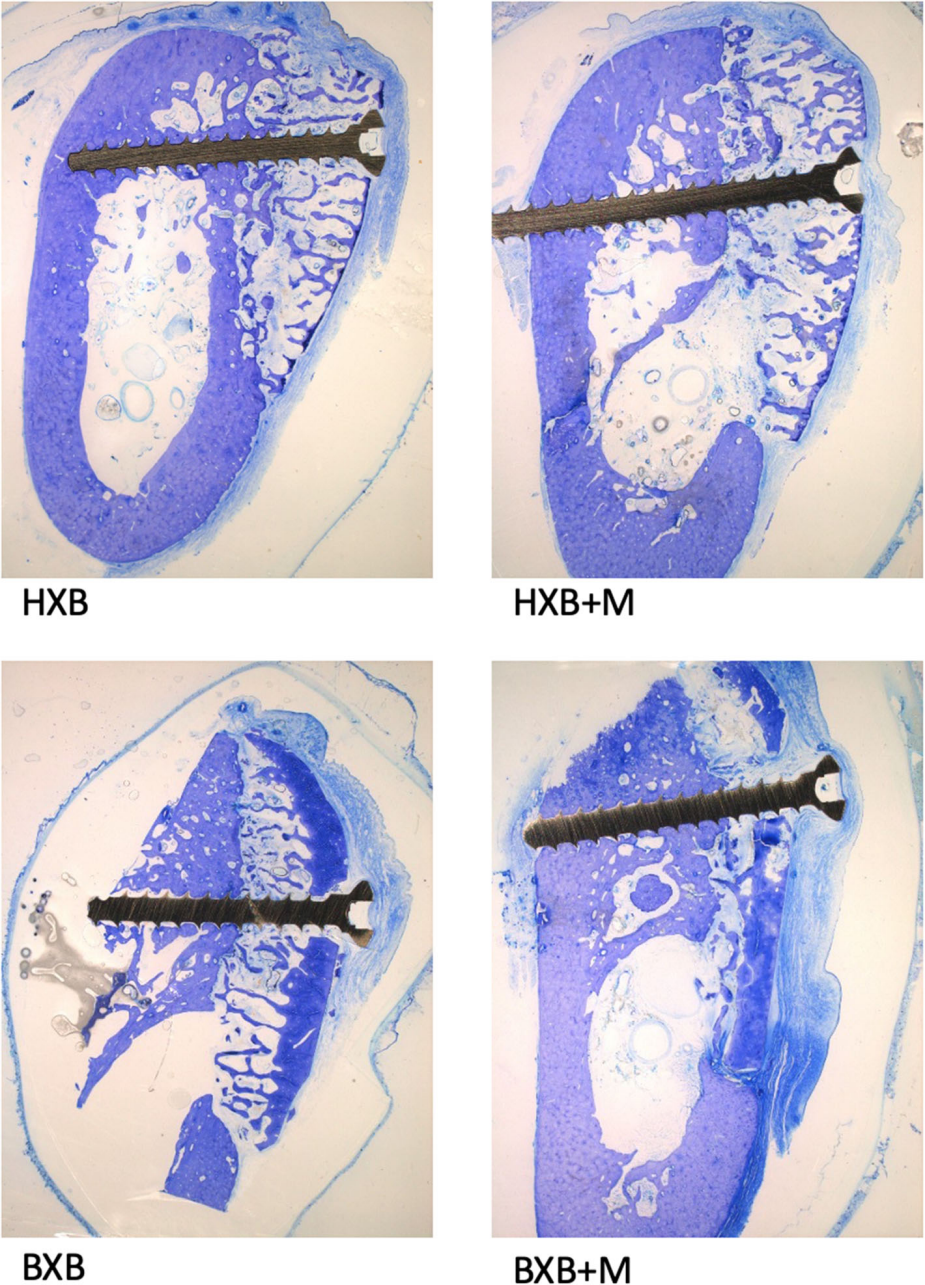
Representative histological cuts for each group. The augmented area is clearly distinguishable. Bovine bone blocks show denser cortical bone. Healing interval of 6 months, toluidine blue, magnification 12.5x

In all samples the augmentation site and the screw used for osteosynthesis were clearly distinguished (Fig. 1). Bovine bone blocks revealed much thicker cortical bone substitute layers on the outline than human bone blocks. Partial fibrous encapsulation of the bone blocks was regularly found. In human bone blocks covered with a collagenous membrane (HXB + M) cancellous new bone was mainly found directly at the borders of residual and augmented bone. No macrophages or giant cells indicating infection were detected. In human bone blocks without a covering membrane (HXB) thin fibrous layers of soft tissue in-between bone trabeculae and soft tissue were found. Little new bone formation was observed and within the bone blocks areas of resorbed bone substitute material were detected. In bovine bone blocks covered with a membrane (BXB + M) a thick cortical layer without bone re-organization was found on the outline of the graft. Bone trabeculae were few and interstitial spaces were filled with collagenous soft tissue. Little new bone formation and areas of resorbed bone substitute material were detected. In some cases, even a collapse of the initial space under the cortical layer could be observed (Fig. 1, BXB + M). Bovine bone blocks without a membranous coverage (BXB) showed equally thick cortical bone. Spaces between bone trabeculae were filled with soft tissue. New bone formation could be solely observed at direct borders of residual bone and substitute material.

In bovine bone blocks in comparison to human bone blocks the augmented area seems condensed and appears to have lost lateral height. This is indicated by the length of the osteosynthetic screw towering the bovine bone blocks after 6 months (Fig. 1, BXB + M).

### Histomorphometry

**Fig. 2:**
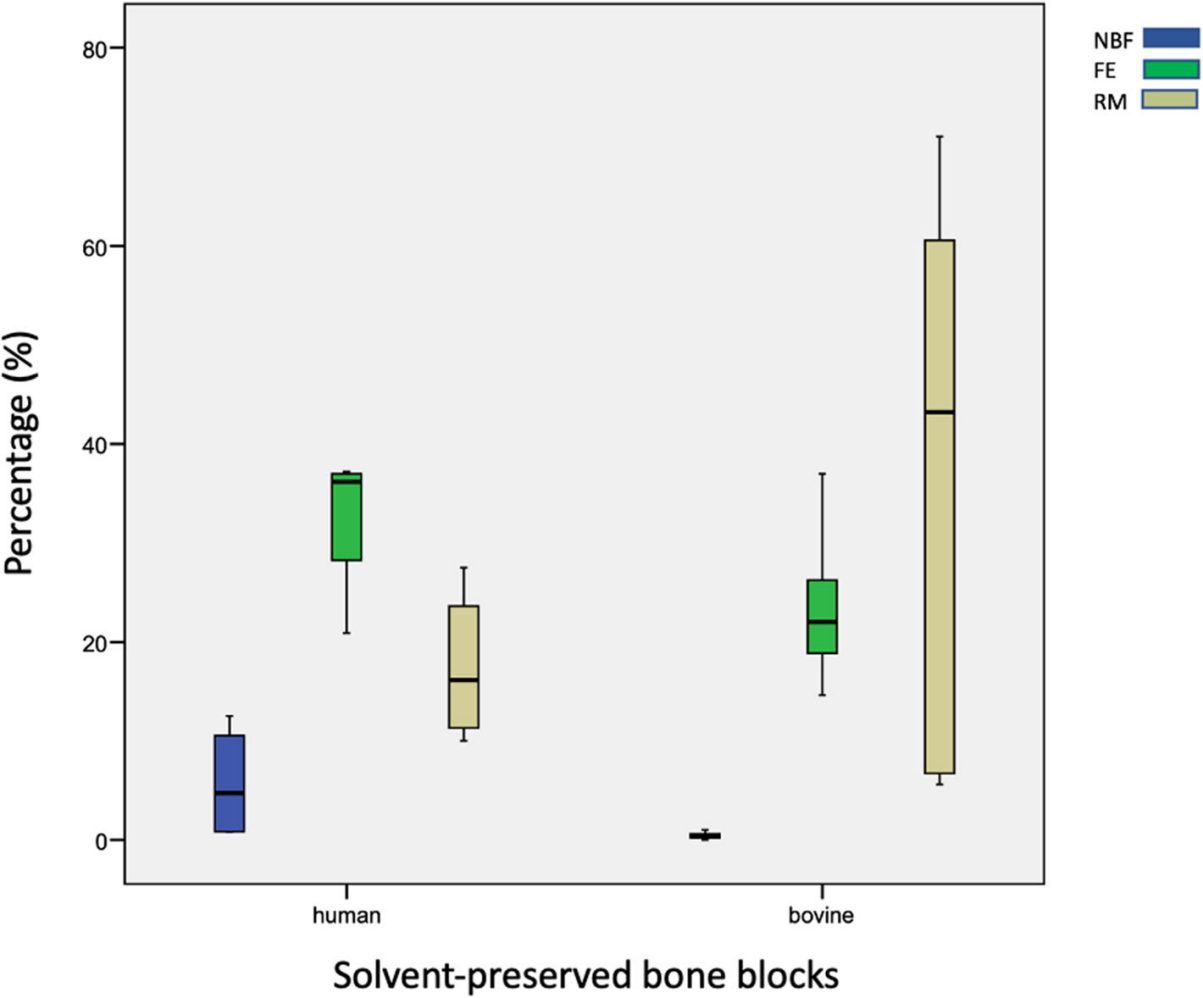
Boxplot diagrams of samples without membrane coverage. Showing percentages of new bone formation (blue), fibrous encapsulation (green), and resorbed matrix (yellow)

**Fig. 3:**
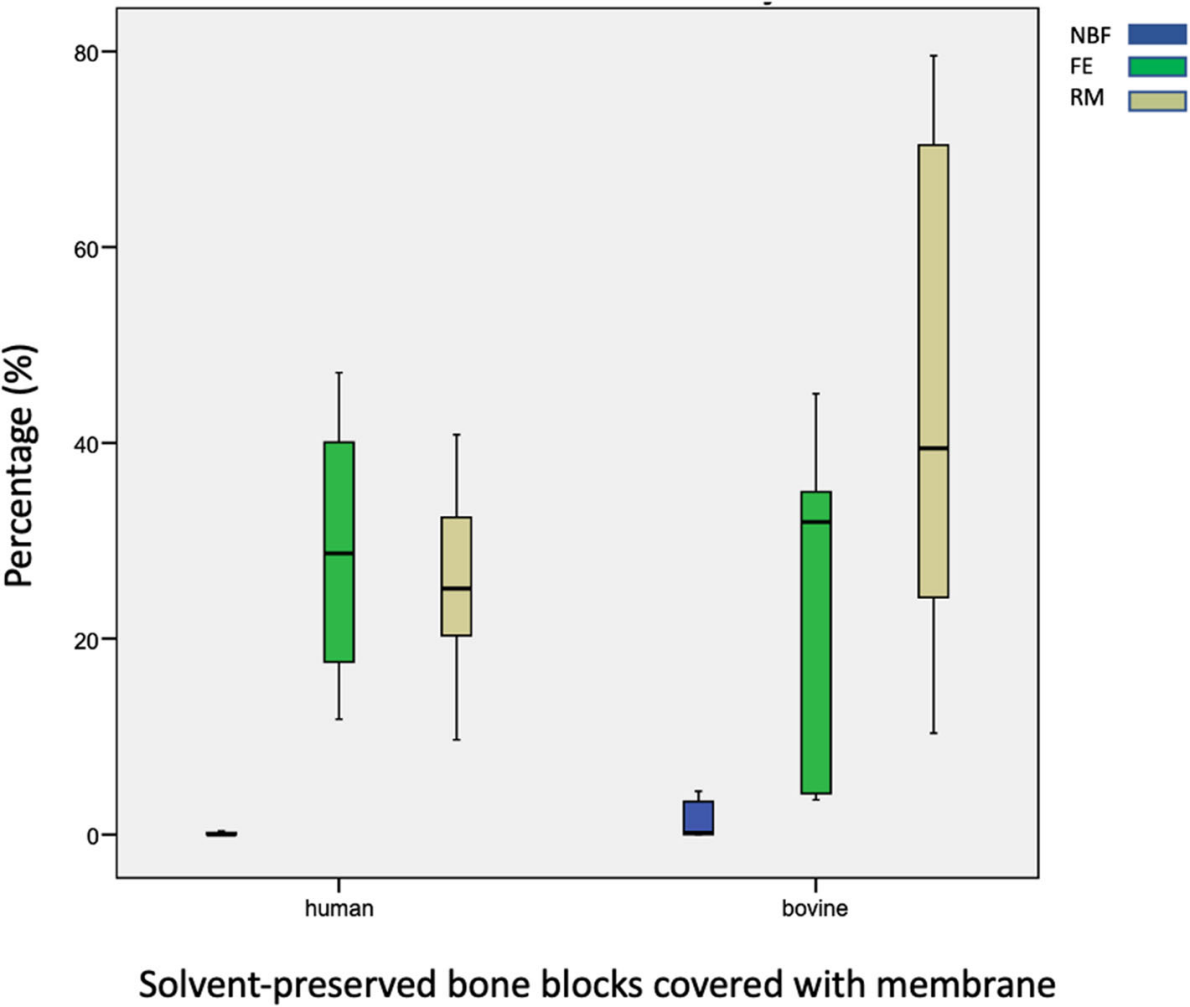
Boxplot diagrams of samples with membrane coverage. Showing percentages of new bone formation (blue), fibrous encapsulation (green), and resorbed matrix (yellow)

All bone blocks showed little new bone formation. HXB showed highest rates of NBF (3.7 % ± 10.2 %). Percentages of NBF in human bone blocks were reduced when covered with a membrane, whereas bovine bone blocks showed higher rates of NBF when covered with a collagenous membrane (HXB vs. BXB *p* = 0.08, HXB vs. HXB + M *p* = 0.09, BXB vs. BXM + M *p* = 0.56, HXB + M vs. BXB + M *p* = 0.12). Fibrous encapsulation was highest in in the HXB + M group (73.71 % ± 10.6 %) and lowest in the BXB + M group (52.5 % ± 28.4 %). Percentages of FE were higher in human bone blocks than in bovine bone blocks (HXB vs. BXB p = 0.98, HXB vs. HXB + M *p* = 0.11, BXB vs. BXM + M *p* = 0.35, HXB + M vs. BXB + M *p* = 0.07. Resorption rates differed from 44.8 % ± 29.6 % in XB + M to 17.4 % ± 7.4 % in HXB. Reduced resorption was detected when bone blocks were covered with a membrane (HXB vs. BXB *p* = 0.24, HXB vs. HXB + M *p* = 0.86, BXB vs. BXM + M *p* = 0.86, HXB + M vs. BXB + M *p* = 0.11). No significant differences were detected. Overall human bone blocks showed more new bone formation and more fibrous encapsulation but reduced resorption in comparison to bovine bone blocks after 6 months (Table 1). Without membranes samples presented similar results (Fig. 2). Membrane-covered human bone blocks showed higher FE but lower RM and NBF than membrane-covered bovine bone blocks (Fig. 3).

**Table 1:** Average percentages of new bone formation, fibrous encapsulation, and resorbed matrix (HXB = solvent-preserved human bone blocks, HXB + M = solvent-preserved human bone blocks covered with a collagenous membrane, BXB = solvent-preserved bovine bone blocks, BXB + M = solvent-preserved bovine bone blocks covered with a collagenous membrane)

	Human		Bovine	
	HXB	HXB + M	BXB	BXB + M
Number (n=)	8	7	6	6
New bone formation (NBF %)	3,7 ± 10,2	0,3 ± 0,4	0,1 ± 0,8	2,6 ± 3,2
Fibrous encapsulation (FE %)	71,2 ± 8,6	73,71 ± 10,6	60,5 ± 27,4	52,5 ± 28,4
Resorption (RM %)	17,4 ± 7,4	25,9 ± 10,7	38,4 ± 27,2	44,8 ± 29,6

## Discussion

In this study lateral bone defects were augmented using bovine and human solvent-preserved cancellous bone blocks. Furthermore, the effect of additional GBR using a collagen membrane was evaluated. Unfortunately, no relevant effects on lateral bone augmentation with or without GBR could be detected. The bone blocks showed high resorption rates, very little new bone formation, and increased fibrous encapsulation.

In general, xenogeneic bone substitutes are suitable for defect regeneration and augmentation. In lateral defects, some authors claim bone substitute materials as gold standard, in particular when used together with dental implants [11]. In literature, they showed similar results to autologous bone in vertical bone regeneration and sinus floor augmentation [16, 17]. In a study by Moon et al. [18], solvent-preserved cancellous bone blocks, in comparison to deproteinized bone blocks, showed significantly more new bone formation in critical size defects in rats [19]. In spinal surgery, however, solvent-preserved bovine cancellous bone blocks were unsuccessfully tested [13]. Autologous bone shows resorption rates of up to 87 % in the lower and 105,5 % in the upper jaw after 6 years when used for augmentation [20]. High resorption rates in this study suggest similar results for solvent-preserved bone blocks. After 6 months human solvent-preserved bone blocks showed 17.4 and 25.9 % resorption, and resorption rates for bovine solvent-preserved bone blocks were at 38.4 and 44.8 %. Bone structures of dogs are more similar to human bone structures than to bovine bone. This might have enhanced new bone formation and reduced resorption. However, fibrous encapsulation was also higher in human bone blocks than in bovine bone blocks, indicating some kind of rejective process.

**Fig. 4:**
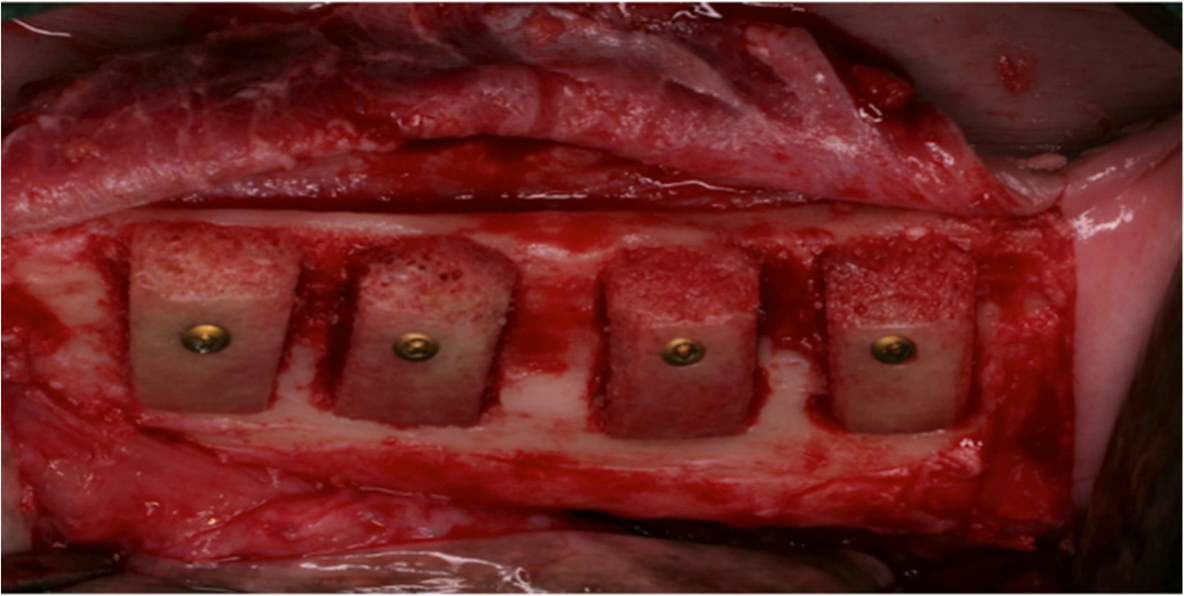
Intraoperative image of the defect augmentation using solvent-preserved human bone blocks (HXB, size 15 × 10 × 6 mm). The bone blocks are fixed with a single screw for osteosynthesis

The main limitation of this study is the lack of a real control group and the small sample size. We compared the augmentative effect to the empty defect before augmentation. Autologous bone blocks, being the current gold standard for lateral defect augmentation, would have been a real control group. In order to use as few animals as possible, the sample size was kept to a minimum. Another limitation of this study is the use of xenogeneic bone blocks. Human and bovine bone blocks used in dogs are of xenogeneic origin. The results cannot be directly transferred onto humans. Bone blocks of human origin would be declared as allogeneic bone blocks and therefore might lead to different results. Xenogeneic materials present altered immunologic characteristics. Immunologic responses to the only partially deproteinized bone blocks might have hindered new bone formation and facilitated fibrous encapsulation. Bone regeneration may benefit from allogeneic, only partially deproteinized materials. A study by Keith et al. [21] showed promising results in vertically augmented bone defects when allogeneic solvent-preserved material was used. Another reason for unsatisfactory results in this study might be defect geometry. The defects solvent-preserved bone blocks were tested on earlier showed almost perfect defect geometries for bone regeneration [18, 21]. In sinus floor augmentation using partially deproteinized allogeneic resorption rates of only around 20 % were seen after 2 years [22]. Solvent-preserved bone blocks might be similar to autologous bone in structure and severity for lateral bone augmentation. They also may be weakened by immunologic degeneration processes. Although in this study the lateral three-walled defects provided good conditions for augmentation, the bone blocks exceeded the defect size by at least 3 mm (eventually more due to remodeling), offering single-walled conditions in the exceeding part (Fig. 4). More durable materials than the materials used might have been necessary. Deproteinized xenogeneic bone blocks, for example, are considered to present excellent volume stability [23]. Against this background some authors suggest choosing augmentative materials according to defect geometry [19]. Fixture by one screw only might have not been enough to ensure rotation stability. Slight movements might have compromised bone regeneration further. The form of application might also influence the outcome. Bone blocks seemed most durable. However, in comparison to a particulate material, the use of solid blocks may hinder angiogenesis and cell immigration due to their dense structure, consequently impeding new bone formation. The thick cortical bone in bovine solvent-preserved bone blocks in particular, therefore, might have been disadvantageous. This thesis is supported by the fact that GBR did not significantly enhance results. On the other hand, angiogenesis is more likely to evolve out of residual alveolar bone than out of the surrounding soft tissue.

The use of membranes in human bone blocks resulted in lower NBF, higher FE, and increased RM. In bovine bone blocks it led to higher NBF, lower FE but higher RM. Results were not significant and contradictory. Immunologic reactions towards the membranes or unnecessary additional barriers for tissue formation might be reasons for its limited effect.

## Conclusions

In conclusion, within the limitations of this study, solvent-preserved xenogeneic bone blocks cannot be recommended for augmentation of lateral bone defects. The use of additional GBR did not affect results. Further research has to follow in order to find suitable options for lateral bone augmentation and to further explore indications for solvent-preserved bone substitutes.

## Data Availability

The datasets used and analyzed during the current study are available from the corresponding author on reasonable request.
